# High-Precision Corrosion Detection via SH1 Guided Wave Based on Full Waveform Inversion

**DOI:** 10.3390/s23249902

**Published:** 2023-12-18

**Authors:** Jiawei Wen, Can Jiang, Hao Chen

**Affiliations:** 1 Institute of Acoustics, Chinese Academy of Sciences, Beijing 100190, China; wenjiawei@mail.ioa.ac.cn (J.W.); chh@mail.ioa.ac.cn (H.C.); 2 School of Electronic, Electrical and Communication Engineering, University of Chinese Academy of Sciences, Beijing 100049, China

**Keywords:** full waveform inversion, tomography, SH1 mode wave, guided wave

## Abstract

Corrosion detection for industrial settings is crucial for safe and efficient operations. Due to its high imaging resolution, the guided–wave full–waveform inversion tomography technique has significant potential for corrosion detection of plate metals. Limited by the long wavelengths of A0 and S0 mode waves, this method exhibits inadequate detection resolution for the earlier shallow and small corrosion defects. Based on the relatively short wavelength characteristics of the SH1 mode wave, we propose a high–precision corrosion detection method via SH1 guided wave using the full waveform inversion algorithms. By conducting finite element simulations of ultrasonic–guided waves on aluminum plates with varying corrosion defects, a comparison was made to assess the detection precision across A0, S0, and SH1 modes. The comparison results showed that, whether for regular or irregular defects, the SH1 mode wave always exhibited higher imaging accuracy than the A0 and S0 mode waves for shallow and small–sized defects. The corresponding experiments were conducted on an aluminum plate with simple or complex defects. The results of the experiments reconfirmed that the full waveform inversion method using SH1 guided wave can effectively reconstruct the shape and size of small and shallow corrosion defects within aluminum plates.

## 1. Introduction

Thin plate structures play a pivotal role in contemporary industry and aerospace sectors, enjoying widespread utilization in aircraft, automobiles, architectural constructions, steel structures [1], energy facilities, and other engineering applications. However, these structures are notably vulnerable to corrosion due to the intricate interplay of environmental and operational factors. The susceptibility to corrosion substantially threatens both safe production and economic benefits, which is particularly accentuated in demanding working environments [2]. Considering these challenges, the development of an accurate and efficient methodology for corrosion detection and evaluation has become crucial [3].

There are several quantitative non–destructive testing (NDT) techniques for the inspection of corrosion in these structures, such as eddy current testing [4], magnetic flux leakage techniques [5], and ultrasonic thickness–gauging methods [6]. All of these methods are based on point–by–point thickness measurements. For the inspection areas with restricted access, such as supports, under coatings or clamps, these methods are difficult to implement without additional effort. What is more, these methods have low inspection efficiency when inspecting large workpieces. The ultrasonic–guided wave detection leverages lower–frequency signals possessing wavelengths commensurate with structural wall thickness and capable of propagating longer distances. This characteristic renders it suitable for long–distance inspection of pipelines and large–area inspection of flat structures [7]. As a technique within guided wave detection, guided wave tomography (GWT) attains high–resolution imaging of internal material defects and structures by analyzing the dispersion attributes of recorded waveforms. This approach is distinguished by its non–invasive nature, high precision, and adaptability to multiple mode waves [8]. Common GWT imaging methods include travel–time tomography [9,10], diffraction tomography [11], the Hybrid Algorithm for Robust Breast Ultrasound Tomography (HARBUT) [12,13], full waveform inversion (FWI) tomography [14,15], and the deep learning imaging method [16].

Compared to other tomographic imaging methods, the full waveform inversion (FWI) method offers superior imaging resolution by effectively incorporating waveform arrival times, phases, and amplitudes, even when utilizing a minimal amount of data. It facilitates multi–parameter modeling and comprehensively accounts for complex acoustic wave phenomena during predictive data simulation [17,18]. This method is widely used in industrial NDT for both bulk wave and guided wave inspection. For bulk wave detection, it has successfully identified delamination in concrete slabs [19], detected cracks in turbine blades [20], located inclusions in gears [21], and delineated delamination in multilayer materials [22,23]. In guided wave detection, Rao J. et al. [24] achieved the high–resolution reconstruction of metallic aluminum plate wall thickness using A0 mode wave for both numerical and experimental models. Compared to other quantitative imaging methods, the FWI method significantly improves the imaging resolution [25]. Additionally, it enabled accurate quantitative imaging of defects in a liquid–loaded plate using an S0 guided wave [26]. Ratassepp M. et al. [27] developed a forward method for describing guided wave propagation in anisotropic plates, achieving quantitative imaging of internal defects in composites using an A0 guided wave. Lin M. et al. [28] employed the A1 mode guided wave with the FWI method to detect complex defects in aluminum plates, with superior accuracy compared to the A0 mode.

Currently, guided wave detection based on the FWI method mainly uses A0 and S0 modes [24,26]. Although this method can achieve imaging accuracy of 1.5~2 wavelengths [25], the A0 and S0 modes have low imaging accuracy in the detection of shallow and small–sized corrosion defects. This limitation is attributed to their frequency band and wavelength. In contrast, the SH1 mode possesses distinct phase velocity dispersion characteristics, featuring a higher frequency and shorter wavelength, which enables it to deliver superior resolution in imaging. Zimmermann. et al. [29] verified that the SH1 mode based on the HARBUT has a higher imaging accuracy compared to the Lamb wave from theoretical simulations to experiments. Meanwhile, Chen et al. [30] effectively employed the SH1 mode with the travel–time tomography to successfully detect blind–hole defects in aluminum plates. Despite these advancements, the conclusive demonstration of quantitative detection of the SH1 mode wave utilizing the FWI algorithm has not been realized in practice.

To address the above issues, we propose the SH1 GWT method using the FWI. Compared with the traditional FWI–GWT method with A0 and S0 modes, the SH1 mode based on FWI has higher imaging accuracy in detecting shallow and small–size corrosion defects. The rest of this paper is organized as follows. The Section 2 introduces the basic principle of the FWI method and the error analysis method. The Section 3 presents the imaging results of numerical simulation models, with a primary focus on comparing the detection accuracy of the A0, S0, and SH1 mode waves for different defects with varying widths and shapes. The Section 4 validates the method by the experiments on a defective aluminum plate. Finally, the Section 5 is a summary.

## 2. Methods

### 2.1. Frequency Domain FWI

**Figure 1:**
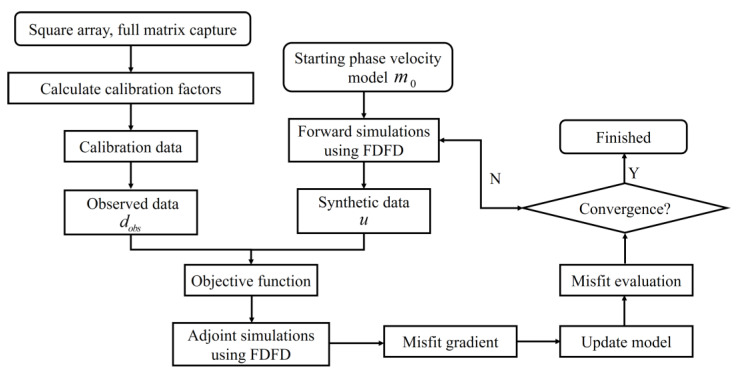
Flow diagram of the GWT algorithm based on FWI.

The core process of the FWI method is to progressively minimize the error function between observed data and computed data through the iterative optimization method. The method can be roughly divided into two parts: the forward process, which aims to solve the acoustic wave equation and simulate the theoretical wave field, and the inversion process, which involves calculating the gradient of the objective function and updating the model parameters through an optimization method. In the inversion process, high sensitivity of the received signals to model parameters is required to guarantee the accuracy of the inversion method, meaning that a small perturbation in the model parameters should induce noticeable changes in the received signals. In this paper, we chose the phase velocity as the model parameter to be inverted. The flow diagram of the FWI–GWT is illustrated in Figure 1.

#### 2.1.1. The Forward Simulation of Guided Wave Propagation

In the gradient calculation process of FWI, for each source, two forward simulations are required: one for source–to–receiver propagation and the other for backward propagation of the residual between the observed data and simulation data. Therefore, the efficiency of the forward simulations significantly impacts the efficiency of the whole process. It is common practice to employ the Helmholtz equation for approximating the guided waves’ propagation along the thin plate. The frequency–domain representation of the Helmholtz equation is as follows:

(1) $$\left(\nabla^{2}+k^{2}\right)u(x,y,\omega )=-f(\omega )\delta (x-x_{0})\delta (y-y_{0})$$

where $\nabla^{2}=\frac{\partial }{\partial x^{2}}+\frac{\partial }{\partial y^{2}}=\partial_{x}^{2}+\partial_{y}^{2}$ signifies the Laplace operator in Cartesian coordinates; $k=\frac{\omega }{v}$ is the wave number, which is related to the angular frequency ω and the speed of the ultrasonic wave v; u(x,y,ω) represents the displacement field; f(ω) denotes the source signal; $\delta (x-x_{0})\delta (y-y_{0})$ represents the Dirac function; and $\left(x_{0},y_{0}\right)$ denotes the source position. The finite difference method is used in the forward simulation process in this study [31]. In order to mitigate interferences caused by the boundary reflections, absorptive layers are implemented at the boundaries to absorb acoustic wave energy, which only produces a minimal increase in computational cost. The discretized form of Equation (1) is represented in matrix notation as follows:

(2) A(x,y,ω)u(x,y,ω)=s(x,y,ω)

where A presents a sparse impedance matrix at a certain frequency ω, and u and s denote the displacement field and the source term, respectively. When the memory meets the requirements of the calculations, the optimal choice is to employ a direct solution based on the sparse LU decomposition to solve Equation (2). The matrix A in Equation (2) can be expressed as

(3) A=LU

where L and U denote the lower and upper triangular matrices of the LU decomposition, respectively. This approach allows for the calculation of wave fields from different sources using the LU decomposition of matrix A, thereby significantly reducing the computational cost.

As the above forward modeling process uses the acoustic wave equation to approximate the propagation of guided waves in the experimental model, the correction factors need to be introduced to reduce disparities between the two models [24]. The expression for the correction factor Q is given as follows:

(4) $$Q=\frac{fft(u_{0})}{fft(d_{obs,0})}$$

where $d_{obs,0}$ represents the time–domain signals that do not pass through any defects during the experimental measurements, $u_{0}$ represents the time–domain signals propagated in a scatheless plate simulated using the finite–difference method, and fft() denotes the fast Fourier transform of the physical quantity inside the parentheses.

#### 2.1.2. Inversion Theory

Let $d_{obs}(\omega ,x_{r},x_{s})$ represent the wavefield obtained from the experimental measurements and $u(\omega ,x,x_{s};v)$ represent the wavefield obtained through numerical simulations. $x_{s}$ and $x_{r}$ denote the location of the source and receivers, respectively, which is restricted to specific positions. Hence, there is a requirement for an operator R to represent the simulated wavefield $Ru(\omega ,x,x_{s};v)=u(\omega ,x=x_{r},x_{s};v)$ at the finite receiver locations. The data residual can be written as $Ru(\omega ,x,x_{s};v)-d_{obs}(\omega ,x_{r},x_{s})$. The objective function using the L2 norm of the residual is defined as follows:

(5) $$E(v)=\frac{1}{2}{\sum_{x_{s}}\sum_{x_{r}}\left[Ru(\omega ,x,x_{s};v)-d_{obs}(\omega ,x_{r},x_{s})\right]}^{2}$$

Due to the independence of the constraint operators R and observed data $d_{obs}(\omega ,x_{r},x_{s})$ from the model, the variation of the objective function can be expressed as follows:

(6) $$\delta E(v)=\frac{1}{2}\delta (Ru-d_{obs},Ru-d_{obs})=(R\delta u,Ru-d_{obs})$$

The variation of the forward wavefield operator δu can be expressed as follows:

(7) $$\delta u=\underset{\varepsilon \to 0}{\lim}\frac{u(v+\varepsilon \delta v)-u(v)}{\varepsilon }$$

In the above equation, both u(v) and u(v+εδv) satisfy Equation (1). Let $u_{\varepsilon }=u(v+\varepsilon \delta v)$ and substitute it into Equation (1):

(8) $$\nabla^{2}u_{\varepsilon }+\frac{\omega^{2}}{{\left(v+\varepsilon \delta v\right)}^{2}}u_{\varepsilon }=s(r,\omega )$$

Based on the linearization assumption, expanding Equation (8) at v using the Taylor expansion method and considering the first–order term:

(9) $$\left(\nabla^{2}+\frac{\omega^{2}}{v^{2}}\right)\delta u=\frac{2\omega^{2}u\delta v}{v^{3}}$$

Equation (9) represents the expression of the acoustic wave equation, which describes the wavefield generated by the source term on the right–hand side. Using Β to represent the forward operator, Equation (6) can be written as

(10) $$\delta E(v)=\left[B\left(\frac{2\omega^{2}u\delta v}{v^{3}}\right),R^{*}(Ru-d_{obs})\right]=\left\{\frac{2\omega^{2}u\delta v}{v^{3}},B^{*}[R^{*}(Ru-d_{obs})]\right\}$$

The second part of the inner product operation in Equation (10) represents the backward propagation of the residual wavefield across the entire model space, which is equivalent to taking the conjugate of the residual wavefield in the frequency domain. Finally, the gradient of the objective function concerning the velocity model is as follows:

(11) $$g(v)=\frac{\delta E(v)}{\delta v}=-\frac{2\omega^{2}}{v^{3}}\sum_{x_{s}}uB^{*}[R^{*}(Ru-d_{obs})]$$

After solving the gradient of the generalized function to the velocity model by setting the initial velocity model, the velocity model needs to be updated based on the optimization algorithm. In this study, the limited–memory Broyden–Fletcher–Goldfarb–Shanno (L–BFGS) algorithm [32] was employed. Compared to the steepest descent method, this algorithm utilizing limited storage space to approximate the inverse Hessian matrix can enhance the computational accuracy and maintain efficiency.

During the updating process of model parameters, it is essential to establish a convergence criterion to terminate the iterative procedure. To avoid classifying noise as defects during the iterations, a convergence criterion based on the difference between the results of each iteration and the results of the last iteration is utilized. The convergence condition α is as follows:

(12) $$\alpha^{\left(n\right)}=\frac{\int \left|d^{n}(x,y)-d^{n-1}(x,y)\right|dxdy}{d_{health}\int dxdy}$$

where $d_{health}$ represents the thickness of the plate without defects, and $d^{n}(x,y)$ denotes the thickness at the *n*–th iteration. The iteration terminates when $\alpha \leq 2\times 10^{-4}$ is reached in this study. Additionally, to prevent the iteration from getting stuck in an infinite loop, the iteration process stops when the number of iterations exceeds M. Based on empirical findings, a loop count number of M = 30 was chosen, which can often yield an optimal inversion result.

### 2.2. Error Analysis

The global relative error is used to assess the reconstruction accuracy. The expression of the global relative error is as follows:

(13) $$E_{global}=\frac{{\left\|\tilde{d}-d\right\|}_{2}}{{\left\|\tilde{d}\right\|}_{2}}$$

where $\tilde{d}$ is the true thickness and d is the thickness reconstructed by the FWI. A smaller global relative error corresponds to a more accurate reconstruction of the defects.

## 3. Numerical Tests

**Figure 2:**
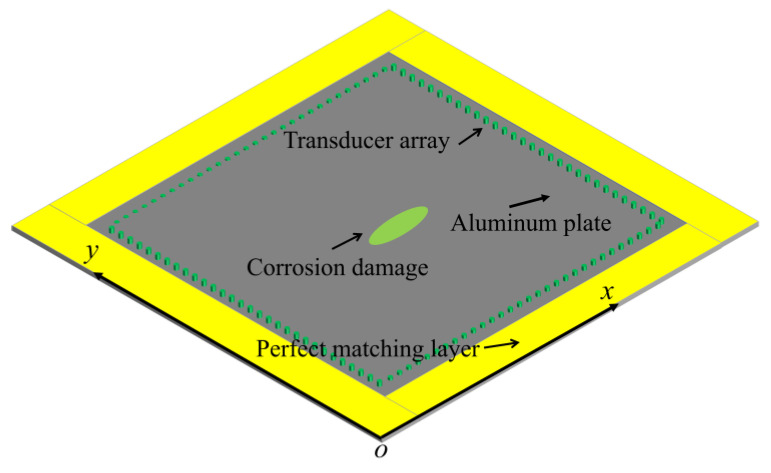
Schematic of the geometric model of an aluminum plate with corrosion damage. There were a total of 120 transducers arranged in a square array on the upper surface.

To compare the reconstruction accuracy of the FWI method using the A0, S0, and SH1 mode waves, we conducted a series of simulations on aluminum plates with different–size defects using COMSOL finite element software (COMSOL Multiphysics^®^ v. 6.0., cn.comsol.com, accessed on 1 July 2023, COMSOL AB, Stockholm, Sweden). The schematic diagram of the model is shown in Figure 2. The grey–filled area represents the aluminum plate. Its density was 2700 kg/m^3^, elastic modulus was 70 GPa, and Poisson’s ratio was 0.33. The green dots on the aluminum plate represent a square transducer array. The length of the side was 400 mm. Each side of the square had 30 transducers, spaced at intervals of 13 mm, totaling 120 transducers on the upper surface of the plate. The yellow area is the perfect matching layer placed around the perimeter of the aluminum plate to reduce the impact of boundary–reflected waves. To obtain the desired guided wave mode and suppress the interferences of other mode waves, the dual–source excitation method was used in the modeling [33]. Two of the same transducers were placed on both the upper and lower surfaces of the aluminum plate. The two transducers were excited by oppositely or identically phased voltages to generate relatively pure A0, S0, or SH1 guided waves in the plate. Therefore, there was a total of 240 transducers on the upper and lower surfaces.

**Figure 3:**
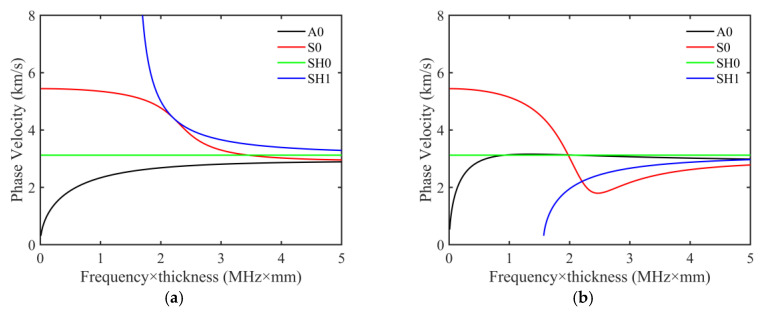
The dispersion curve for an aluminum plate. (**a**) Phase velocity. (**b**) Group velocity.

Figure 3a,b display the phase and group velocity dispersion curves, respectively, of A0, S0, SH0, and SH1 mode waves for an aluminum plate. The frequency–thickness products ranged from 0 to 5 MHz × mm. As can be seen from Figure 3a, the phase velocity of the SH0 mode wave remained unchanged with the variation of the frequency–thickness products, meaning that this mode is insensitive to thickness information and is unsuitable for plate thickness inversion. In contrast, the phase velocity of A0, S0, and SH1 modes displayed notable changes in the frequency–thickness product ranges of 0.35~0.5, 1.5~1.75, and 2.25~2.5 MHz × mm, respectively. Consequently, for a 10 mm thick aluminum plate, the excitation frequencies for the above three modes were selected as 50, 175, and 250 kHz, respectively. The corresponding wavelengths for these modes were 37.6 mm, 33.4 mm, and 16 mm, respectively. According to Huthwaite [13], the maximum transducer spacing is recommended to be less than half a wavelength. However, deploying an excessive number of transducers in practical experiments is not feasible. Consequently, transducer spacing is usually set within one wavelength [24]. For the three mode waves, the selected spacing of 13 mm between adjacent transducers satisfies the fundamental requirements.

### 3.1. Regular Defect

To more easily describe the size and position of the defect for an aluminum plate, a thickness shape function for the defect was introduced:

(14) $$d(x)=\left\{\begin{array}{c} d_{0}-\frac{d}{2}\left[1+\cos\left(\frac{\left(\left|x\right|-0.8r\right)}{r-0.8r}\right)\right]\\ d_{0}-h\\ d_{0} \end{array}\begin{array}{c} ,0.8r<|x|<r\\ ,|x|\leq 0.8r\\ ,|x|>r \end{array}\right.$$

where $d_{0}$ represents the plate thickness before corroding, set as 10 mm in this study. The maximum corroded depth *h* was set as 1 mm to assess the reconstructing accuracy of the FWI method using A0, S0, and SH1 mode waves. The defect was modeled as an inverted trapezoidal cylinder with upper and lower radii of *r* and 0.8*r*, respectively. The seamless transition between these surfaces was accomplished by a half–period cosine function. The center of the defect is located at the geometric center of the transducer array. By adjusting the parameter *r*, we can obtain the simulation data for the aluminum plate with different–size defects using the finite element method.

**Figure 4:**
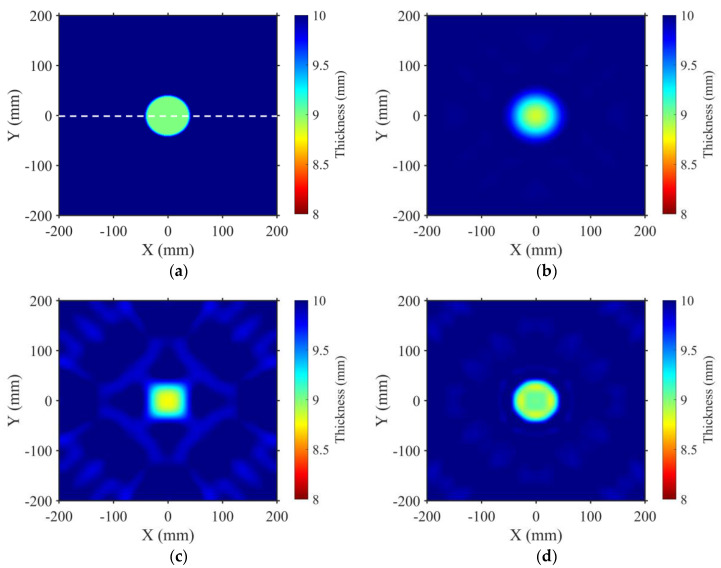
Reconstructed thickness maps using the A0, S0, and SH1 mode waves for a circular defect (*r* = 45 mm). (**a**) True thickness map. The white dashed line indicates the location for extracting profile thickness distribution maps in Figure 5g. (**b**) Thickness map reconstructed by the A0 mode wave. (**c**) Thickness map reconstructed by the S0 mode wave. (**d**) Thickness map reconstructed by SH1 mode wave.

**Figure 5:**
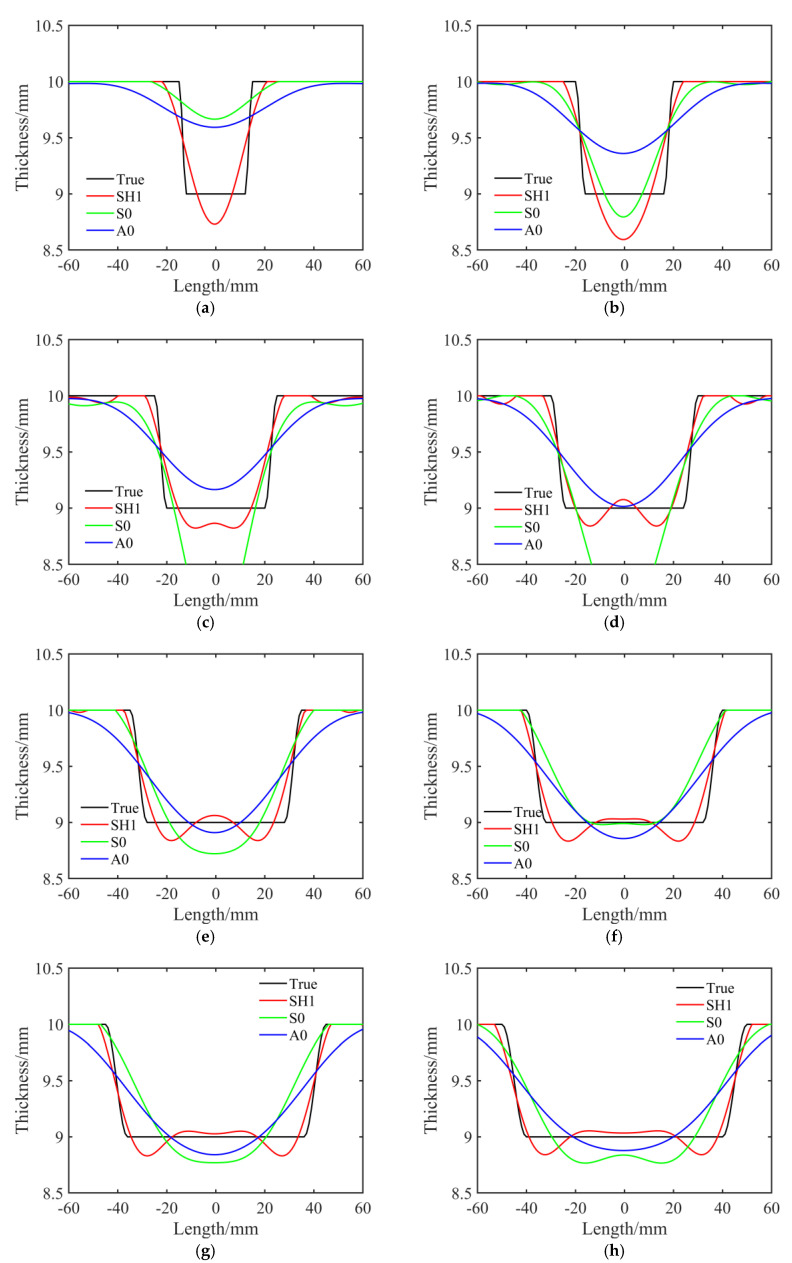
Reconstructed thickness distributions using the A0, S0, and SH1 mode waves for an axisymmetric round defect with radii ranging from 15 mm to 50 mm. The black line represents the true thickness distribution of the profile at the *y* = 0 mm axis, while the red, green, and blue lines correspond to the inversion results using the SH1, S0, and A0 mode waves, respectively. (**a**) *r* = 15 mm. (**b**) *r* = 20 mm. (**c**) *r* = 25 mm. (**d**) *r* = 30 mm. (**e**) *r* = 35 mm. (**f**) *r* = 40 mm. (**g**) *r* = 45 mm. (**h**) *r* = 50 mm.

Figure 4 illustrates the reconstruction results of a defect with a radius of 45 mm (*r* = 45 mm, *h* = 1 mm) using the FWI method. The true thickness map is displayed in Figure 4a. The white dashed line represents the cross–section for detailed comparisons of the imaging thickness distributions between the true model and reconstruction results. Figure 4b–d display the reconstructed thickness maps utilizing A0, S0, and SH1 mode waves, respectively. From the imaging results, it became apparent that all three modes displayed certain artifacts in their reconstruction maps, stemming from the inherent multi–solution nature of the inversion problem. For the shape and size reconstructions of the defect, especially the detailed depiction of the outline, the SH1 mode wave exhibited the most favorable performance compared with the A0 and S0 mode waves. To further assess the reconstruction accuracy of the defect thickness, Figure 5g presents the thickness distribution of the profile along the dashed line in Figure 4a. It can be observed that the SH1 mode provided a higher precise reconstruction of the defect depth and width, while the S0 mode wave tended to overestimate the defect depth, and the A0 mode wave tended to overestimate the defect size.

To thoroughly investigate the reconstruction accuracy of the three mode waves under different–size defects, we adjusted the defect size parameter *r* in Equation (14) and simulate eight sets of simulation data. The maximum corroded depth *h* was also set as 1 mm. The radii of these circular defects ranged from 15 mm to 50 mm, with intervals of 5 mm. The reconstructed profile thickness distributions along the *y* = 0 mm axis for the aluminum plates with various defect sizes are shown in Figure 5. As can be seen from Figure 5, limited by the long wavelength, the A0 mode waves always overestimated the sizes of the defects and were unable to delineate the edge shapes of the defects. Unlike the A0 mode wave, the S0 mode wave showed some advantages in the size and depth evaluations of the defects. However, with the decrease in the defect sizes, especially when the radius of the defect was less than its wavelength, the S0 mode wave was unable to acquire the true depth of the defects. Compared with the A0 and S0 mode waves, the SH1 mode wave had considerable advantages in the size, edge shape, and depth evaluations of defects because of its short wavelength and high sensitivity to plate depth.

To quantitatively assess the reconstruction accuracy of the A0, S0, and SH1 mode waves, we calculated the global relative errors (Equation (13)) for defects with various sizes. As shown in Table 1, no matter whether a small defect or a big defect, the SH1 mode wave exhibited the lowest global relative error relative to the A0 and S0 mode waves, which further demonstrates its highest imaging accuracy.

### 3.2. Irregular Defect

**Figure 6:**
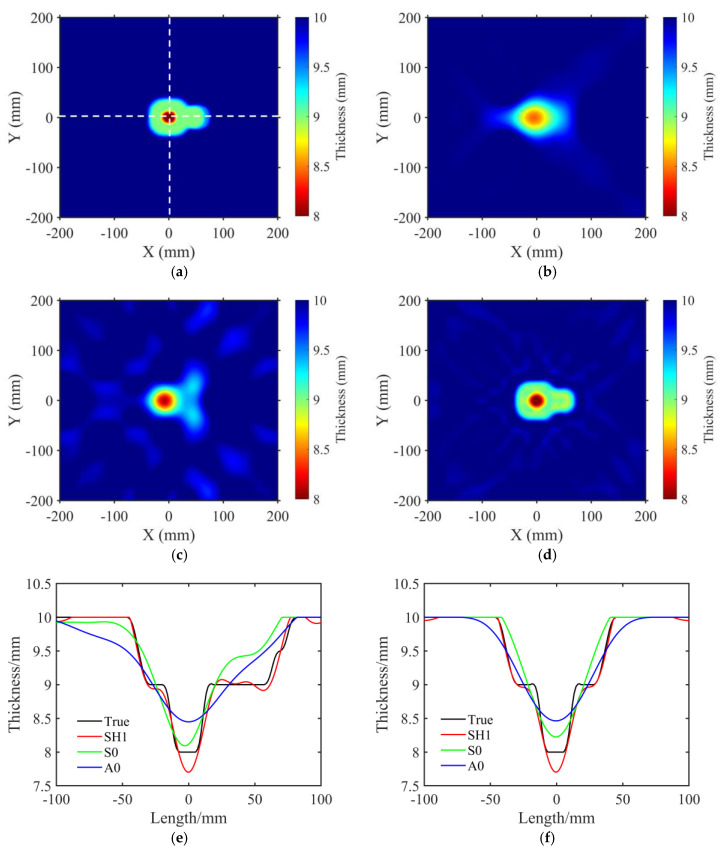
Reconstructed thickness maps using the A0, S0, and SH1 mode waves for the irregular defect. (**a**) True thickness map. The white dashed line indicates the location for extracting profile thickness distribution maps. (**b**) Thickness map reconstructed by A0 mode. (**c**) Thickness map reconstructed by S0 mode. (**d**) Thickness map reconstructed by SH1 mode. (**e**) Thickness distribution along the white horizontal dashed line in (**a**). The black line represents the true thickness distribution of the profile, while the red, green, and blue lines correspond to the inversion results obtained using the SH1 mode, S0 mode, and A0 mode, respectively. (**f**) Thickness distribution along the white vertical dashed line in (**a**), with the same notations for the inversion results as in (**e**).

In consideration of irregularly shaped defects and variations in corrosion thickness that may be encountered in practical scenarios, we established the following model. The thickness map of the model is shown in Figure 6a. The model primarily consisted of two defects with different corrosion depths. One was a rounded rectangle, whose geometric center was located at (0 mm, 0 mm). The side length was 80 mm. The corrosion depth was 1 mm. The center of this rectangle was also corroded by a small, rounded rectangle with a side length of 30 mm. The corrosion depth was 2 mm. The side length of another rounded rectangle defect was 50 mm. The geometric center was located at (50 mm, 0 mm). The corrosion depth was 1 mm. The full–matrix data acquisition was accomplished by the same squared array in Section 3.1.

Figure 6b–d illustrates the reconstructed thickness maps for the A0, S0, and SH1 mode waves, respectively. It can be seen that both A0 and S0 mode waves failed to image the shape and size of the irregular defect. Only the SH1 mode wave successfully reconstructed both the 1 mm depth shallow defect and the 2 mm depth small–sized defect, showcasing the remarkable precision of the SH1 mode in detecting shallow and small–sized defects. The detailed comparison of the thickness distributions along two orthogonal directions (white dash line in Figure 6a) is given in Figure 6e,f, respectively. The black line represents the real thickness distribution. Unlike the A0 (blue line) and S0 (green line) mode waves, the SH1 mode wave (red line) effectively reconstructed the size, edge shape, and depth evaluations of defects. The well–fit results demonstrate the accuracy of the imaging result of the SH1 mode wave. Table 2 shows the global relative error of the three mode waves. The SH1 mode exhibited the lowest global relative error, further explaining its high imaging accuracy.

## 4. Experiment Tests

To further validate the accuracy of the SH1 mode in practical defect detection, we set up a corresponding experiment. The experimental setup comprised a computer, a signal generator, an oscilloscope, and a power amplifier. The experiment was conducted on a 6061–aluminum plate with a three–dimensional size of 1000 mm × 1000 mm × 10 mm. Its density was 2700 kg/m^3^. The elastic modulus was 70 GPa and the Poisson’s ratio was 0.33. A total of 120 d15 piezoelectric sensors were used to form a square array, with 30 sensors on each side. The spacing between two adjacent ones was 13 mm. The dimensions of the d15 piezoelectric sensor were 12 mm × 6 mm × 0.8 mm. The piezoelectric sensors were affixed on the aluminum plate using the 502 adhesive. In contrast to the simulations in Section 3, the actual inspection process is often limited to installing sensors on only one surface due to convenience considerations, especially in the inspection of pipelines and spherical tanks. Therefore, in our experiment, piezoelectric sensors were only mounted on one side of the aluminum plate.

**Figure 7:**
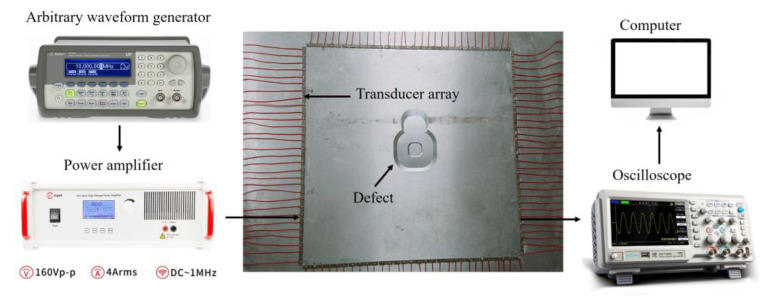
Experimental configuration and aluminum plate.

**Figure 8:**
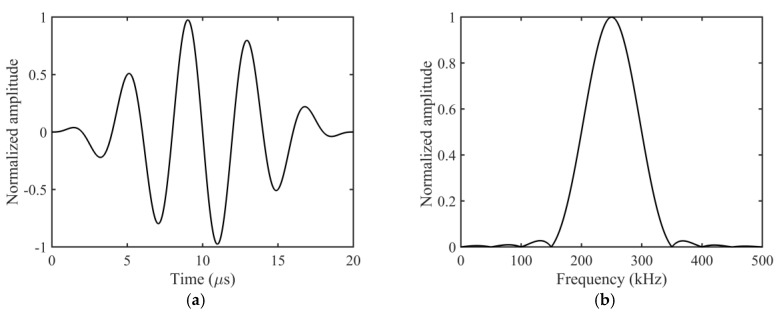
Excitation signal with a center frequency of 250 kHz. (**a**) The waveform in time domain. (**b**) The corresponding normalized amplitude spectrum.

The structure of the experimental setup is illustrated in Figure 7. During the experiment, the signal generator generated a source signal, which was amplified by the power amplifier to excite the transmitting transducer. The ultrasonic–guided wave signals propagated along the plate were received by the receiving transducer and acquired by the oscilloscope. The data acquired by the oscilloscope were further processed on the computer. To excite the SH1 mode with a larger amplitude while avoiding the generation of the higher–order mode, a five–cycle Hanning window modulated signal with a central frequency of 250 kHz was selected as the source signal. The time–domain waveform and amplitude of the source signal are shown in Figure 8a,b, respectively. On each side of the square array, the 30 sensors were sequentially employed as transmitters to generate waveforms, while the 30 sensors on the opposing side were used as receivers to capture waveform data. By repeating this procedure, we ultimately obtained 30 × 30 × 4 channels of waveform data.

### 4.1. Data Preprocessing

**Figure 9:**
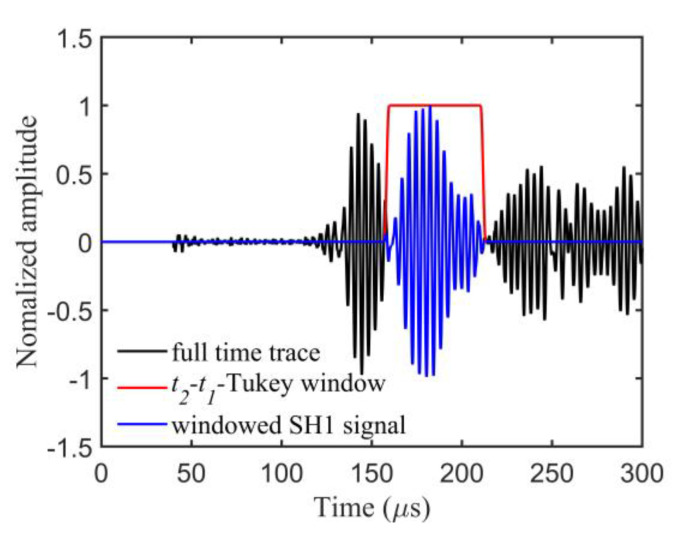
The waveform acquired from a transmitter–receiver pair in the experiment. The black line denotes full–time trace. The red line denotes the Tukey window based on *t*_1_ and *t*_2_. The blue line denotes the windowed SH1 signal.

The acquired signals were often accompanied by noise signals. A bandpass filter based on the bandwidth of the transducer was first used to eliminate the noise signals. Figure 9 displays the received waveform (black line) from one transmitter–receiver pair. Different from the simulated data, exciting and receiving an SH–type guided wave on one side of the plate often results in the acquired signals containing a prominent SH0 mode wave before the arrival of the SH1 guided wave (blue line in Figure 9). Based on the analysis in Section 3, the SH0 mode wave has no sensitivity to the thickness variation and cannot provide available thickness information. The waveform that arrived following the SH1 guide wave is the reflected waves generated by the edges of the plate and the defects in the plate. Although the reflected waves carry partial information about the defects, effectively extracting the defects’ information is a difficult task. In this paper, only the direct wave (SH1 mode wave) was used in the inversion. The Tukey window method, as described in [29], was used for the extraction of the SH1 mode wave from the received waveforms. Utilizing the group velocity dispersion curves of SH0 and SH1 mode waves, we established the starting point $t_{1}$ and ending point $t_{2}$ of the time window as follows:

(15) $$t_{1}=\frac{s_{n}}{c_{g,\mathrm{SH}0}}+0.7\times (\frac{s_{n}}{c_{g,\mathrm{SH}1}}-\frac{s_{n}}{c_{g,\mathrm{SH}0}})$$

(16) $$t_{2}=1.3\times \frac{s_{n}}{c_{g,\mathrm{SH}1}}$$

where $c_{g,\mathrm{SH}0}$ and $c_{g,\mathrm{SH}1}$ represent the group velocity of the SH0 and SH1 mode waves, respectively, and $s_{n}$ denotes the distance of the *n*–th transmitter–receiver pair. The red line in Figure 9 denotes the Tukey window based on the parameters $t_{1}$ and $t_{2}$, and the blue line represents the SH1 mode wave obtained after the Tukey window processing. Following the Fourier transformation of the SH1 mode wave data, we proceeded to apply the frequency–domain FWI method for the defects imaging.

### 4.2. Regular Defect

**Figure 10:**
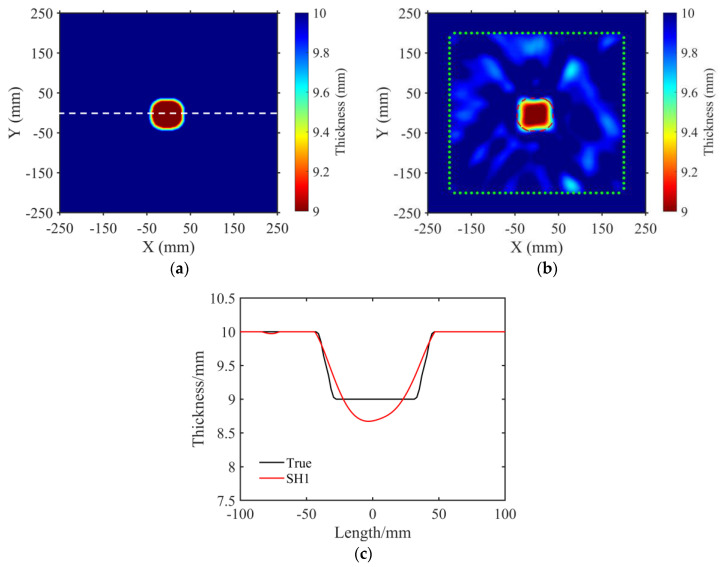
Reconstructed thickness maps using the SH1 mode wave for the regular defect. (**a**) True thickness map. The white dashed line indicates the location for extracting profile thickness distribution maps. (**b**) Thickness map reconstructed by SH1 mode. The red dashed line represents the outer contour of the actual model, while the green dots denote the transducer locations. (**c**) Thickness distribution along the white horizontal dashed line in (**a**). The black line represents the true thickness distribution of the profile, while the red line corresponds to the reconstructed results obtained using the SH1 mode.

Similar to the process of simulation experiments, a rounded rectangle defect was fabricated on the upper surface of the aluminum plate through the CNC milling method. The side length of the defect was 80 mm. The depth of the cutting face was 1 mm. The true plate thickness map of the defective aluminum plate is shown in Figure 10a. The black solid line in Figure 10c illustrates the profile depth at the *y* = 0 mm axis (white dashed line in Figure 10a). The data acquisition was accomplished using the above–mentioned experimental equipment and measuring process. Figure 10b displays the imaging result for the defective aluminum plate using the FWI method. The red dashed line represents the outline of the rounded rectangle defect. It can be seen that the shape and position of the defect were accurately reconstructed. The slight deformation of the reconstructed round rectangle defect may have been caused by the position error of the square array and the consistency of each sensor arising from the adhesive process. Figure 10c displays the reconstructed thickness distribution (red line) of the profile at the *y* = 0 mm axis. The maximum error of the estimated defect thickness was 0.3 mm. The well–fitting result between the reconstructed and actual thickness distributions demonstrated the accuracy of the imaging method. The global relative error of 0.43% for the reconstruction of the regular defect further illustrates the high accuracy of the method.

### 4.3. Irregular Defect

**Figure 11:**
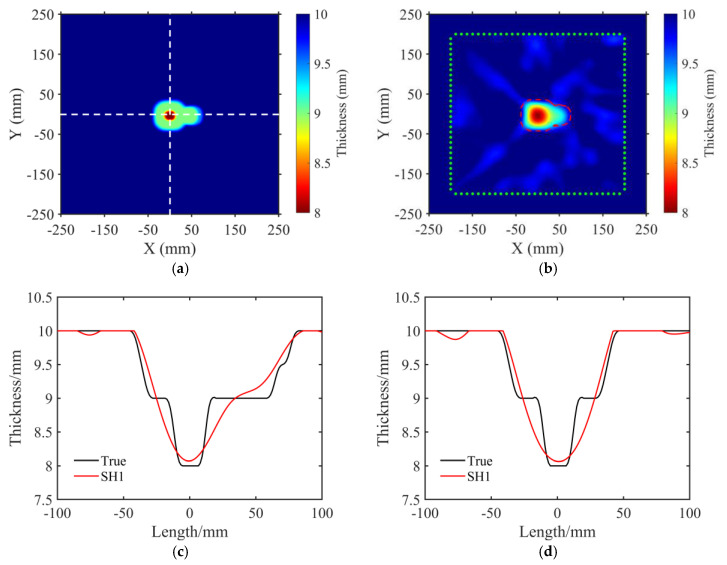
Reconstructed thickness maps using the SH1 mode for an irregular defect. (**a**) True thickness map. The white dashed line indicates the location for extracting profile thickness distribution maps. (**b**) Thickness map reconstructed by SH1 mode. The red dashed line represents the outer contour of the actual model, while the green dots indicate the transducer placement locations. (**c**) Thickness distribution along the white horizontal dashed line in (**a**). The black line represents the true thickness distribution of the profile, while the red line corresponds to the inversion result obtained using the SH1 mode. (**d**) Thickness distribution along the white vertical dashed line in (**a**), with the same notations for the inversion results as in (**c**).

To test the imaging capacity for irregular defects that usually occur in practical corrosion processes, we further processed the defective aluminum plate to create a complex and irregular defect. The shape and size of the irregular defect were consistent with the model in Figure 6a. The real thickness map of the model is also shown in Figure 11a. The profile thickness of this model at the *y* = 0 mm axis is presented in Figure 11c as a black line, while the profile thickness at the *x* = 0 mm axis is shown in Figure 11d as a black line. Based on the FWI method, we provide the imaging result of the irregular defect using the SH1 mode wave in Figure 11b. The red dashed line represents the outline of the irregular defect. As the figure shows, the imaging result displayed a good reconstruction for the left rounded rectangle (side length was 80 mm, depth of cross–section was 1 mm). Conversely, for the right (side length was 50 mm, depth of cross–section was 1 mm) and the center (side length was 30 mm, and depth of cross–section was 2 mm) rounded rectangle, the reconstruction capacity of the outlines slightly decreased. For further analysis, the reconstruction thickness distributions at the *y* = 0 mm and *x* = 0 mm axis are given in Figure 11c, d, respectively. The maximum error for the maximum corrosion depth was less than 0.1 mm.

Table 3 provides the global relative error of the irregular defect reconstruction. The error using the SH1 mode wave in the experimental process was greater than that observed in the simulations. The experimental model closely mirrored the simulation model. This discrepancy can be attributed to factors such as random noise, fewer transducers, poor transducer consistency, position error, and the slight anisotropy of the material in the actual measurement process. Similarly, the experimental reconstruction errors of A0 and S0 were larger than the simulation under the same modeling [24,26]. Compared with the imaging result in Figure 6, we found that the reconstruction of the shape and outline for the experimental defects using the SH1 mode wave was more accurate than that using A0 and S0 mode waves in the simulation. Furthermore, the imaging errors for irregular defect and the reconstruction error observed in the experimental result using the SH1 mode wave were smaller than that using the A0 and S0 modes in the simulation. Since the reconstruction error of the experiment was necessarily larger than that of the simulation case, this indicated that under the same experimental conditions, the reconstruction error of the SH1 mode was necessarily smaller than that of the A0 and S0 modes. Therefore, it can be confidently stated that the SH1 mode, based on the FWI method, delivers higher imaging accuracy than the A0 and S0 modes for actual shallow surface defect detection. The method proposed in this paper can provide a new nondestructive testing method for practical shallow and small defect detection.

## 5. Conclusions

Based on the FWI algorithm, we propose a method for detecting corrosion defects within metal plates using the SH1 ultrasonic guided wave. Finite element simulation data indicated that this method exhibits superior imaging precision than that using the A0 and S0 mode waves, making it particularly suitable for shallow and small–sized defect detection. The experimental results revealed that, whether for regular or irregular defects in shape, this method can accurately reconstruct the shape and location of the corroded area, providing a reliable method for the practical inspection of shallow and small–sized defects.

It is noteworthy that, during practical inspections, an in–plane excitation method is imperative for the generation of the SH1 mode wave, owing to its inherent in–plane vibration characteristics. In comparison to the A0 and S0 mode waves, the currently employed excitation method exhibits lower efficiency, rendering it more apt for inspection tasks characterized by short distances and smaller dimensions. Furthermore, the SH1 mode manifests a low–frequency cutoff frequency and susceptibility to influence from the SH2 mode wave within the high–frequency band. Consequently, its applicability is confined to the detection of defects within a specific range of thickness variations (≤20% nominal thickness).

Subsequent research will explore the guided–wave mode separation method, aiming to enhance the thickness detection range of the SH1 mode wave. In addition, the propagation characteristics of guided waves in the plate are similar to those in the pipe when the wall thickness is far less than the cylinder diameter and the wavelength is smaller than or comparable to the pipe wall thickness [34]. Currently, there are relatively few studies on pipeline detection using SH1 mode, and corresponding research will be carried out in the future.

## Figures and Tables

**Table 1:** The global relative errors in defect reconstruction for various size defects using the A0, S0, and SH1 mode waves (data from simulation).

*r*	*E_global_*		
	A0	S0	SH1
15 mm	0.70%	0.42%	0.26%
20 mm	0.74%	0.27%	0.26%
25 mm	0.77%	0.92%	0.23%
30 mm	0.81%	0.92%	0.30%
35 mm	0.82%	0.67%	0.33%
40 mm	0.86%	0.57%	0.37%
45 mm	0.74%	0.81%	0.34%
50 mm	0.61%	0.55%	0.31%

**Table 2:** The global relative errors in irregular defect reconstruction using the A0, S0, and SH1 mode waves (data from simulation).

Mode	*E_global_*
A0	0.46%
S0	0.64%
SH1	0.14%

**Table 3:** The global relative errors in defect reconstruction using the SH1 mode wave (data from the experiment).

Defect Category	*E_global_*
Regular defect	0.43%
Irregular defect	0.41%

## Data Availability

Data underlying the results presented in this paper are not publicly available at this time but may be obtained from the authors upon reasonable request.
